# Pretreatment blood biomarkers combined with magnetic resonance imaging predict responses to neoadjuvant chemoradiotherapy in locally advanced rectal cancer

**DOI:** 10.3389/fonc.2022.916840

**Published:** 2022-08-09

**Authors:** Xinyu Shi, Min Zhao, Bo Shi, Guoliang Chen, Huihui Yao, Junjie Chen, Daiwei Wan, Wen Gu, Songbing He

**Affiliations:** ^1^ Department of General Surgery, The First Affiliated Hospital of Soochow University, Suzhou, China; ^2^ Department of Nuclear Medicine, The First Affiliated Hospital of Soochow University, Suzhou, China

**Keywords:** locally advanced rectal cancer, neoadjuvant chemoradiotherapy, pathological complete response, blood biomarkers, magnetic resonance imaging, prognosis

## Abstract

**Aim:**

To investigate the value of pretreatment blood biomarkers combined with magnetic resonance imaging (MRI) in predicting the efficacy of neoadjuvant chemoradiotherapy (NCRT) in patients with locally advanced rectal cancer (LARC).

**Methods:**

This study involved patients with LARC who received NCRT and subsequently underwent total mesenteric excision from June 2015 to June 2021 at the First Affiliated Hospital of Soochow University. Patients with incomplete courses of neoadjuvant therapy, comorbidities with other malignancies or diseases that affect the study outcome, and those who underwent unplanned surgery were ultimately excluded. Laboratory data such as albumin, CEA, various blood cell levels, and MRI related data such as tumor regression grade assessed by magnetic resonance imaging (mrTRG) were collected from the included patients one week prior to NCRT. MrTRG is a common clinical imaging metric used to assess the degree of tumor regression in rectal cancer, primarily based on morphological assessment of residual tumor. Furthermore, pretreatment blood biomarkers such as neutrophil to lymphocyte ratio (NLR), lymphocyte to monocyte ratio (LMR), albumin to fibrinogen ratio (AFR), and prealbumin to fibrinogen ratio (PFR) were assessed. The independent variables for pathologic complete response (pCR) to NCRT were determined by univariate and multivariate logistic regression analyses. Receiver operating characteristic (ROC) curve analysis was used to examine the performance of MRI with or without pretreatment blood biomarkers in predicting pCR using DeLong’s method. A nomogram was created and confirmed internally.

**Results:**

Fifty-nine individuals with LARC satisfied the inclusion criteria, among which 23 showed pCR after NCRT. Logistic regression analysis demonstrated that pretreatment CEA (≤ 3 µg/L, OR = 0.151, P = 0.039), NLR (OR = 4.205, P = 0.027), LMR (OR = 0.447, P = 0.034), and PFR (OR = 0.940, P = 0.013) were independent predictors of pCR to NCRT. The AUCs of mrTRG alone and mrTRG plus the above four pretreatment blood biomarkers were 0.721 (P =0.0003) and 0.913 (P <0.0001), respectively. The constructed nomogram showed a C-index of 0.914.

**Conclusion:**

Pretreatment blood biomarkers combined with MRI can help clinical efforts by better predicting the efficacy of NCRT in patients with locally advanced rectal cancer.

## Introduction

According to the global cancer statistics in 2020, colorectal cancer (CRC) is the second deadliest malignant tumor worldwide (1). Rectal cancer accounts for approximately 30% of colorectal cancer cases, and the proportion is increasing annually. In addition, most patients with rectal cancer are already locally advanced at the time of diagnosis and have a poor prognosis.

Since studies have reported the superiority of preoperative chemoradiotherapy over postoperative chemoradiotherapy, the conventional treatment modality of locally advanced rectal cancer (LARC) is neoadjuvant chemoradiotherapy (NCRT) followed by total mesenteric excision (TME) (2, 3). In recent years, the total neoadjuvant therapy (TNT) modality and the watch-and-wait (W&W) strategy have received increasing attention (4–7).

However, a significant variation in individual responses to NCRT has been noted during clinical treatment. Approximately 50%-60% of rectal cancer patients show staged shrinkage after NCRT, whereas about 10%-30% show pathologic complete response (pCR) (5). However, approximately one-third of patients show poor sensitivity to chemoradiotherapy, and NCRT efficacy is strongly linked to the prognosis of these patients (8). Therefore, the early prediction of NCRT efficacy is particularly important in the diagnosis and treatment of locally advanced rectal cancer.

Based on the principles of pathological tumor regression grading (pTRG), Patel et al. proposed magnetic resonance imaging for assessing tumor regression grade (mrTRG) in 2011 (9). However, this traditional morphological qualitative assessment based on T2-weighted imaging may fail to predict treatment response when assessing residual tumors (10). Since conventional MRI provides only morphological information, it is difficult to distinguish treatment-induced fibrosis, necrosis, and tumor residuals (11). In contrast, functional MRI such as diffusion-weighted imaging (DWI) can provide information at the molecular level of the tumor (12). DWI indirectly reflects the biology of human tissues by assessing the diffusive motion of water molecules and providing a quantitative index of the apparent diffusion coefficient (ADC). Recent studies have used DWI techniques to assess the efficacy of neoadjuvant therapy in patients with rectal cancer (13, 14). The efficacy of ADC values in predicting the efficacy of neoadjuvant therapy for rectal cancer remains controversial. The reasons for this may be related to factors such as the use of different methods to outline the region of interest (ROI) and different b-values. Therefore, until a uniform standard is reached in clinical as well as scientific research, assessment based on a single imaging image is inevitably a bit subjective.

Several economically feasible blood markers have been explored in recent clinical studies to predict tumor regression response after NCRT, such as neutrophil to lymphocyte ratio (NLR), prognostic nutritional index (PNI), and carcinoembryonic antigen (CEA) (15–23). Some foreign scholars explored whether the combined use of magnetic resonance imaging (MRI) parameters with CEA levels could better predict the efficacy of NCRT than MRI parameters alone. It was found that the combination of mrTRG and CEA improved the AUC value from 0.680 to 0.728 compared to mrTRG alone (24). However, although the performance of MRI parameters in combination with CEA for predicting pTRG improved, it was still unsatisfactory. Therefore, it was natural to question whether more satisfactory results could be obtained using additional and more valuable blood biomarkers in combination with MRI.

Thus, in this study, we aimed to investigate whether combining multiple blood biomarkers with T2WI-based mrTRG could significantly improve the power of MRI in predicting the efficacy of neoadjuvant chemoradiotherapy in patients with LARC. We also established a new model of MRI parameters and multiple blood markers. We have reason to believe that this is the first study to combine multiple blood biomarkers with MRI to predict the efficacy of neoadjuvant chemoradiotherapy in rectal cancer. This study will provide new ideas and methods for the selection of treatment strategies for neoadjuvant chemoradiotherapy in patients with rectal cancer.

## Materials and methods

### Patients

This retrospective study initially screened LARC patients who underwent NCRT and subsequent surgery at the First Affiliated Hospital of Soochow University from June 2015 to June 2021. The follow-up period was from the clinical diagnosis of rectal cancer to 2 weeks after TME surgery, encompassing the entire neoadjuvant treatment. The inclusion criteria were as follows: (1) rectal cancer with positive clinical stage T3-T4 or positive lymph nodes as determined by preoperative MRI, without distant metastases; (2) adenocarcinoma of the rectum less than 10 cm from the anal verge as confirmed by pathology of the colonoscopic biopsy specimen; (3) no previous chemotherapy or pelvic radiotherapy experience; (4) complete clinical process information, including laboratory test results within 7 days before the start of NCRT and tumor pathological characteristics; (5) complete imaging information, including rectal MRI images 4 weeks before NCRT and 6-8 weeks after NCRT; and (6) complete resection without positive tumor margins. The standards for exclusion were as follows: (1) incomplete completion of preoperative chemoradiotherapy treatment; (2) evidence of acute and chronic infections, autoimmune diseases, and hematological disorders; (3) palliative surgery or partial resection or emergency surgery; and (4) synchronous malignancies or medical history of other malignancies. This study was approved by the ethics committee of the First Affiliated Hospital of Soochow University.

### Treatment

In this study, all patients received neoadjuvant chemoradiotherapy. Patients received preoperative radiation in the pelvic region in 25 fractions at a dose of 45 Gy, and the original tumor was irradiated with an additional 5.4 Gy in three doses, making the maximum dosage 50.4 Gy (25). Capecitabine was administered at a dose of 825 mg/m^2^ twice daily from Monday to Friday throughout the radiotherapy period. In the interval after radiotherapy and before surgery, patients received 2 to 3 cycles of neoadjuvant chemotherapy in one of two regimens, the CapeOX (43 cases, 72.9%) and the FOLFOX (16 cases, 27.1%). All patients underwent surgery according to the principle of TME at 4 to 8 weeks after NCRT. Patients were considered for adjuvant chemotherapy 3–4 weeks following surgery.

### Pathological assessment of the response to NCRT

Pathological response to NCRT was evaluated by two independent pathologists according to the four-tier American Joint Committee on Cancer (AJCC) seventh edition tumor regression grade (TRG) classification. The pathological TRGs (pTRGs) system was defined as follows: pTRG0, no remaining viable cancer cells; pTRG1, single cells or rare residual cancer cells; pTRG2, residual cancer with a desmoplastic response; and pTRG3, minimal evidence of tumor response (26). The pCR was defined as pTRG 0 and the other grades were defined as non-pCR.

### MRI assessment of the response to NCRT

All patients underwent rectal MRI 4 weeks before and 6-8 weeks after NCRT. The assessment of rectal cancer MRI parameters was performed by two radiologists with more than 3 years of experience in rectal cancer MRI staging. T-stage, N-stage, the distance from the anal verge to the lower edge of the tumor, and the status of the circumferential resection margin (mrCRM) were assessed by rectal MRI 4 weeks prior to NCRT. If the distance between the tumor and the mesorectal fascia on MRI was greater than or equal to 1 mm, the case was considered definitive mrCRM (9). The assessment of mrTRG was based on rectal MRI 6-8 weeks after NCRT: grade 1, mucosal or mucosal inferior 1 to 2 mm scar or marked normalization of the rectal wall; grade 2, dense fibrosis with no obvious residual tumor; grade 3, more than 50% of fibrosis or mucus and visible residual tumor signal; grade 4, minimal fibrosis/mucinous degeneration, mostly tumor; and grade 5, same as a primary tumor or tumor progression (10). Like pTRGs, mrTRGs were classified into good response and poor response, with mrTRG 1 or 2 and mrTRG 3, 4, or 5 indicating good and poor response, respectively.

### Data collection and definitions

All patients underwent routine blood tests, liver and kidney function tests, coagulation tests, and serum CEA tests. All blood specimens were tested in our laboratory one week before the start of NCRT. The pretreatment blood biomarkers were calculated as follows:


NLR=neutrophil count /lymphocyte count;

PLR=platelet count /lymphocyte count;

LMR=lymphocyte count /monocyte count;

SII=platelet count×neutrophil count/lymphocyte count;

PNI=10×serum albumin(g/dL)+0.005×total lymphocyte count(per mm3);

AFR=Serum albumin/fibrinogen;

PFR=Serum prealbumin/fibrinogen

In previous studies different cut-off values have been used for these biomarkers. For example, for NLR, Braun LH et al. adopted a cut-off value of 4.06, and neoadjuvant therapy tended to work well in patients with rectal cancer with pre-treatment NLR below 4.06 (15). However, some studies have also used 2.0 and 3.05 as cut-off values for NLR (22, 23). And there are also some scholars who did not convert these biomarkers into dichotomous variables (20, 21). Therefore, our study used continuous variables for all biomarkers.

### Statistical analyses

The Statistical Package for the Social Sciences, version 20.0, was used to conduct statistical analyses (IBM SPSS Inc., Chicago, USA). Continuous variables were analyzed using the Student’s t-test for normally distributed variables or the Mann-Whitney U test for skewed distributed variables. Categorical variables were assessed using the Chi-square test or Fisher’s exact test (if the expected frequencies were <5). A univariate and multivariate logistic regression model was utilized to determine predictive factors for pCR to NCRT. DeLong’s technique was used to compare the areas under the curves (AUC) based on receiver operating characteristic (ROC) curves analysis of mrTRG alone versus the combination of mrTRG and pretreatment blood biomarkers for the prediction of pCR. A predictive nomogram was developed using R version 4.1.3 (R-Project, Institute of Statistics and Mathematics, Vienna, Austria) based on the findings of multivariate logistic regression analysis. The nomogram’s performance was evaluated using internal validation and AUC. Furthermore, the Harrell’s concordance index (C-index) was calculated to evaluate the discriminating capability of the nomogram. A two-sided P < 0.05 was considered statistically significant.

## Results

### Patient characteristics

**Figure 1:**
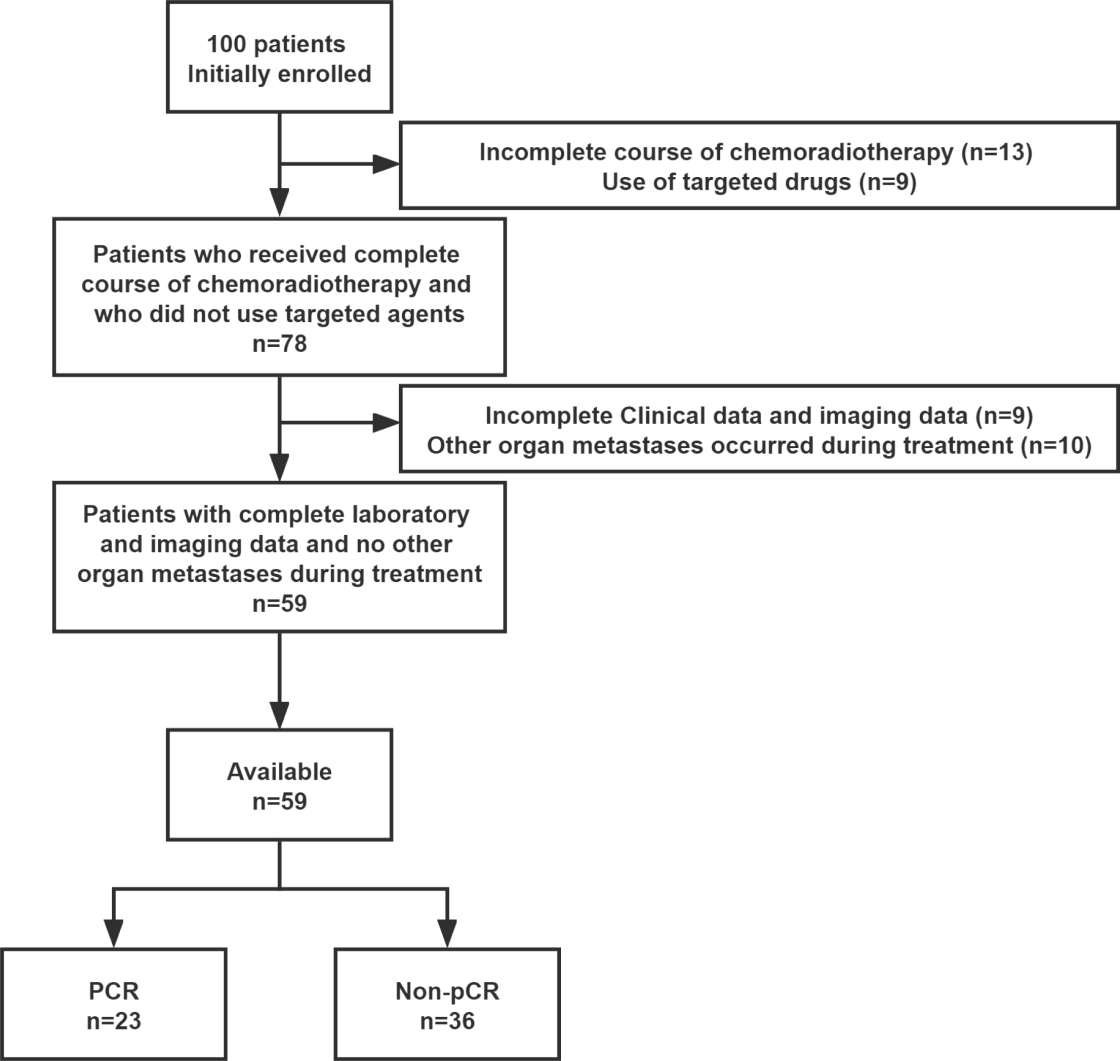
Filtering process of patient data from the initial inclusion of patients.

From June 2015 to June 2021, we initially enrolled 100 LARC patients to receive neoadjuvant therapy, with 59 patients ultimately completing the study (see **Figure 1**). Patients who have not completed their course of chemoradiotherapy (n = 13), those who received concomitant targeted agents during NCRT (n = 9), those with incomplete laboratory records or imaging data (n = 9), and those with metastases to other organs (n = 10), were excluded from the study. Ultimately, 59 patients who satisfied all criteria were included in the study. All patients included underwent rectal MRI for clinical staging and assessment of treatment outcome before and after neoadjuvant chemoradiotherapy. For all 59 patients, the pTRGs according to each mrTRG are displayed in **Table 1**. Patient characteristics are summarized in **Table 2**. Among the 59 patients, pCR (pTRG 0) was observed in 23 (29.0%) patients, pTRG 1 in 16 (27.1%), pTRG 2 in 8 (13.6%) and pTRG 3 in 12 (20.3%). The median pretreatment biomarkers levels of serum albumin, prealbumin, hemoglobin, NLR, PLR, LMR, SII, PNI, AFR, and PFR were 41.1 g/L (range,33.1-49.2), 222.6 mg/L (range,149.9-345.5), 136 g/L (range,73-147), 2.65 (range,1.46-4.55), 133.85 (range,50.23-562.50), 3.59 (range,2.03-9.00), 552.3 (range,241.66-1644.78), 41.11 (range,33.11-49.21), 18.19 (range,14.13-22.17), and 95.95 (range, 60.69-157.05), respectively. The number of patients with CEA >3µg/L was 41(69.5%).

**Table 1:** pTRG according to mrTRG.

mrTRG	pTRG				Total
	0	1	2	3	
mrTRG 1	7 (77.8)	2 (22.2)	0	0	9 (15.3)
mrTRG 2	7 (63.6)	3 (27.3)	1 (9.1)	0	11 (18.6)
mrTRG 3	6 (26.1)	8 (34.8)	4 (17.4)	5 (21.7)	23 (39.0)
mrTRG 4	3 (21.4)	2 (14.3)	3 (21.4)	6 (42.9)	14 (23.7)
mrTRG 5	0	1 (50.0)	0	1 (50.0)	2 (3.4)
Total	23 (39.0)	16 (27.1)	8 (13.6)	12 (20.3)	59 (100.0)

Values are expressed as number (%). pTRG, pathologic tumor regression grade; mrTRG, tumor regression grade assessed by magnetic resonance imaging.

**Table 2:** Patient characteristics and response to NCRT.

Variables	Number (%) (n = 59)	PCR (n = 23)	Non-pCR (n = 36)	*P*
**Gender**				
Male	44 (74.6%)	15	29	0.156
Female	15 (25.4%)	8	7	
**Age**				
≥ 60	30 (50.8%)	12	18	0.542
< 60	29 (49.2%)	11	18	
**BMI**				
≥ 24	27 (45.8%)	8	19	0.139
< 24	32 (54.2%)	15	17	
**Distance from the anal verge (cm)**				
≥ 5	30 (50.8%)	9	21	0.121
< 5	29 (49.2%)	14	15	
**Clinical T stage**				
T 1-2	5 (8.5%)	1	4	0.346
T 3-4	54 (91.5%)	22	32	
**Clinical N stage**				
N 0-1	25 (42.4%)	11	14	0.341
N 2	34 (57.6%)	12	22	
**mrCRM**				
(+)	24 (40.7%)	6	18	0.059
(–)	35 (59.3%)	17	18	
**mrTRG**				
1-2 (Good)	20 (33.9%)	14	6	**0.001**
3-5 (Poor)	39 (66.1%)	9	30	
**Pretreatment biomarkers levels [median (range)]**				
Serum albumin (g/L)	41.1 (33.1-49.2)	41.2 (33.1-49.2)	40.95 (34.9-47.4)	0.196
Serum prealbumin (mg/L)	222.6 (149.9-345.5)	251.5 (165.5-345.5)	215.9 (149.9-332.0)	0.058
Hemoglobin (g/L)	136 (73-147)	134 (73-155)	136 (94-174)	0.217
NLR	2.65 (1.46-4.55)	2.17 (1.46-4.01)	2.74 (1.46-4.55)	**0.011**
PLR	133.85 (50.23-562.50)	155.19 (50.23-562.50)	131.27 (77.1-348.57)	0.232
LMR	3.59 (2.03-9.00)	4.76 (2.16-9.00)	3.38 (2.03-8.61)	**0.005**
SII	552.3 (241.66-1644.78)	488.43 (284.75-1625.63)	598.47 (241.66-1644.78)	0.511
PNI	41.11 (33.11-49.21)	41.21 (33.11-49.21)	40.96 (34.91-47.41)	0.196
AFR	18.19 (14.13-22.17)	18.77 (14.84-22.17)	17.61 (14.13-20.04)	**0.041**
PFR	95.95 (60.69-157.05)	106.08 (75.23-157.05)	92.04 (60.69-144.98)	**0.028**
CEA				
> 3	41 (69.5%)	11	30	**0.007**
≤ 3	18 (30.5%)	12	6	

NCRT, neoadjuvant chemoradiotherapy; pCR, pathologic complete response; BMI, body mass index; MrCRM, mesorectal circumferential resection margin status; MrTRG, tumor regression grade assessed by magnetic resonance imaging; NLR, neutrophil to lymphocyte ratio; PLR, platelet to lymphocyte ratio; LMR, lymphocyte to monocyte ratio; SII, systemic immune-inflammation index; PNI, prognostic nutritional index; AFR, albumin to fibrinogen ratio; PFR, prealbumin to fibrinogen ratio; CEA, carcinoembryonic antigen. Bold values indicate that P-value is significant.

### Predictors of pCR to NCRT

The relationships between patient demographics, tumor features, pretreatment biomarkers and MRI parameters, and pCR are shown in **Table 2**. Clinical biomarkers such as gender, age, BMI, the distance from the anal verge to the lower edge of the tumor, T stage, N stage, and mrCRM, and pretreatment blood biomarkers such as serum albumin, serum prealbumin, hemoglobin, PLR, SII, and PNI were not associated with pCR to NCRT (all P > 0.05).

According to the univariate analysis, mrTRG (1-2 vs. < 3-5, OR = 0.129, 95% CI 0.038-0.432, P = 0.001), pretreatment CEA level (≤ 3.0 vs. > 3.0, OR = 0.183, 95% CI 0.055-0.608, P = 0.006), pretreatment NLR (OR = 2.648, 95% CI 1.202-5.834, P = 0.016), pretreatment LMR (OR = 0.581, 95% CI 0.396-0.851, P < 0.001), pretreatment AFR (OR = 0.674, 95% CI 0.456-0.997, P = 0.048), and pretreatment PFR (OR = 0.969, 95% CI 0.941-0.998, P = 0.036) were significantly associated with pCR to NCRT (**Table 3**). Multivariate Logistic regression analysis demonstrated that mrTRG (1-2 vs. < 3-5, OR = 0.074, 95% CI 0.011-0.499, P = 0.007), pretreatment CEA level (≤ 3.0 vs. > 3.0, OR = 0.151, 95% CI 0.025-0.913, P = 0.039), pretreatment NLR (OR = 4.205, 95% CI 1.175-15.052, P = 0.027), pretreatment LMR (OR = 0.447, 95% CI 0.212-0.939, P < 0.034), and pretreatment PFR (OR = 0.940, 95% CI 0.896-0.987, P = 0.013) were independent predictors of pCR to NCRT (**Table 3**).

**Table 3:** Univariate and Multivariate Logistic regression analysis for response to NCRT.

Variables	Univariate analysis		Multivariate analysis	
	OR (95% CI)	*P*	OR (95% CI)	*P*
**mrTRG**				
1-2 vs. 3-5	0.129 (0.038-0.432)	0.001	0.074 (0.011-0.499)	**0.007**
**Pretreatment CEA (µg/L)**				
≤ 3.0 vs. > 3.0	0.183 (0.055-0.608)	0.006	0.151 (0.025-0.913)	**0.039**
**Pretreatment biomarkers**				
NLR	2.648 (1.202-5.834)	0.016	4.205 (1.175-15.052)	**0.027**
LMR	0.581 (0.396-0.851)	0.005	0.447 (0.212-0.939)	**0.034**
AFR	0.674 (0.456-0.997)	0.048	0.730 (0.379-1.403)	0.345
PFR	0.969 (0.941-0.998)	0.036	0.940 (0.896-0.987)	**0.013**

NCRT, neoadjuvant chemoradiotherapy; mrTRG, tumor regression grade assessed by magnetic resonance imaging; CEA, carcinoembryonic antigen; NLR, neutrophil to lymphocyte ratio; LMR, lymphocyte to monocyte ratio; AFR, albumin to fibrinogen ratio; PFR, prealbumin to fibrinogen ratio. CI, confidence interval. Bold values mean that P-value is significant.

Overall, the pCR group had higher LMR and PFR, but lower NLR and CEA levels.

### Pretreatment biomarkers improve the predictive performance of MRI

**Figure 2:**
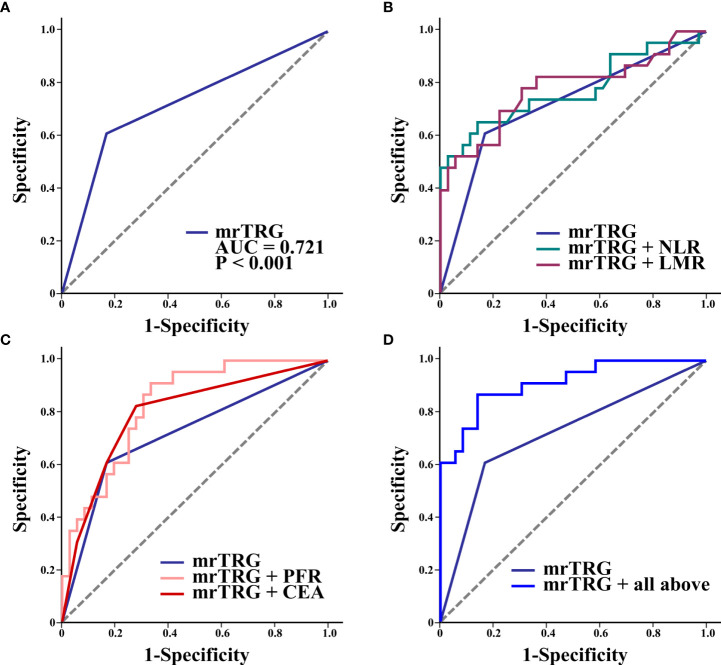
ROC curves of mrTRG (1-2 vs. 3-5) alone **(A)** and mrTRG plus pretreatment biomarkers (NLR, LMR, PFR, CEA and all above four biomarkers) **(B–D)** for the prediction of pCR.

**Figure 2** shows the ROCs for mrTRG alone (**Figure 2A**) and mrTRG plus pretreatment blood biomarkers for predicting pCR (**Figures 2B-D**). The AUCs for mrTRG plus biomarkers for predicting pCR were significantly larger than that for mrTRG alone (**Table 4** **)**.

**Table 4:** AUC values of each roc curve.

Parameters	pCR	
	AUC (95% CI)	P^a)^
mrTRG	0.721 (0.589-0.830)	0.0003
mrTRG + NLR	0.774 (0.647-0.873)	0.0001
mrTRG + LMR	0.778 (0.651-0.876)	<0.0001
mrTRG + PFR	0.831 (0.711-0.916)	<0.0001
mrTRG + CEA	0.798 (0.674-0.892)	<0.0001
mrTRG + all above four biomarkers	0.913 (0.810-0.971)	<0.0001

pCR, pathologic complete response; mrTRG, tumor regression grade assessed by magnetic resonance imaging; CEA, carcinoembryonic antigen; NLR, neutrophil to lymphocyte ratio; LMR, lymphocyte to monocyte ratio; AFR, albumin to fibrinogen ratio; PFR, prealbumin to fibrinogen ratio. CI, confidence interval.

a P are for the comparison of each AUC and that of mrTRG based on DeLong’s method.

### Nomogram for pCR to NCRT

**Figure 3:**
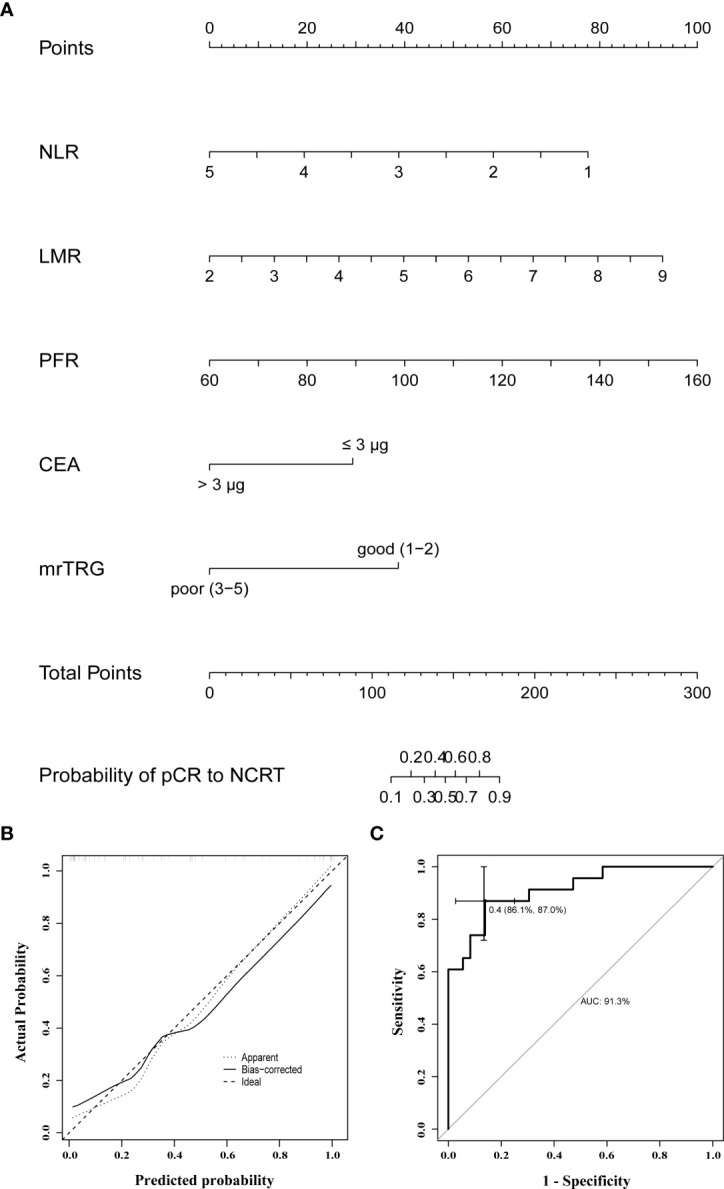
**(A)** A nomogram for predicting the probability of pCR to NCRT in LARC patients; **(B)** curves with internal validation for the nomogram; **(C)** ROC analysis of the nomogram. NCRT, neoadjuvant chemoradiotherapy; pCR, pathologic complete response; mrTRG, tumor regression grade assessed by magnetic resonance imaging; NLR, neutrophil to lymphocyte ratio; LMR, lymphocyte to monocyte ratio; PFR, prealbumin to fibrinogen ratio; CEA, carcinoembryonic antigen.

Based on the significant predictors in the logistic regression analysis, a nomogram for the prediction of pCR to NCRT in LARC patients was developed, as shown in **Figure 3A**. The predicted probability of pCR for NCRT could be easily obtained by adding up the scores of each variable and then drawing a straight line. The patients with higher total scores tended to achieve a higher probability of pCR to NCRT. The internally validated calibration curves revealed good agreement between the predicted and actual probability of pCR to NCRT (**Figure 3B**). The nomogram performance was verified internally, and it exhibited a C-index of 0.914 (95% CI 0.838-0.988) and AUC of 0.913, as illustrated in **Figure 3C**.

## Discussion

The results of this study revealed that 23 (39.0%) of 59 LARC patients who received NCRT achieved pCR. CEA, NLR, LMR, and PFR were significant predictors of pCR, superior to markers such as PLR, SII, and PNI. High values of pretreatment LMR and PFR and low values of NLR and CEA were positively correlated with pCR. High pre-NCRT AFR was positively correlated with pCR in univariate logistic regression analysis, but not in multivariate logistic regression analysis. This discrepancy may be due to the high correlation between PFR and AFR in multivariate regression analysis. In the ROC curves analysis, the AUCs of individual biomarkers (NLR, LMR, PFR, and CEA) in combination with mrTRG were 0.774, 0.778, 0.831, and 0.798, respectively, which were all higher than that of mrTRG alone (AUC of 0.721). Furthermore, expectedly, the AUC of mrTRG in combination with all four biomarkers (NLR, LMR, PFR, and CEA) was the highest (0.913).

Scholars are increasingly recognizing a possible cross-link between systemic inflammatory responses and nutritional risk, as well as tumor-associated immune responses. Various inflammatory cells and inflammatory mediators are important components of the tumor microenvironment. For example, lymphocytes can induce cytotoxicity leading to tumor cell death and inhibit tumor cell proliferation and migration (27, 28). Sustained local and systemic inflammatory responses can be involved in the development, progression and prognosis of many malignancies through various mechanisms such as inhibition of DNA damage and apoptosis by inflammatory cytokines (29, 30). Malignancies can in turn lead to severe nutritional imbalances and even cachexia, directly activating proteolysis and lipolysis in target organs through a variety of pathways, such as pro-inflammatory factors with catabolic effects that can act as mediators of cachexia (31). This catabolism occurs mainly in skeletal muscle, adipose tissue, and myocardium, and its consequences include increased chemotherapy toxicity, complication rates of surgery, and increased mortality (32). In contrast, the cytotoxic effect of chemoradiotherapy causes necrosis of tumor cells and alters the local and systemic inflammatory response, thus increasing the recognition of tumor antigens by the body’s immune system (33). Therefore, early assessment of the sensitivity of patients with malignant tumors to radiotherapy is essential.

In imaging, conventional rectal MRI is a classic tool for clinical assessment of rectal cancer staging and the effectiveness of neoadjuvant therapy. The application of apparent diffusion coefficient (ADC) values from diffusion-weighted imaging (DWI) to predict the tumor regression response after NCRT in rectal cancer has been studied (34–36). Some studies have investigated the performance of maximum standardized uptake values (SUVs) of 18F-FDG PET or its dynamics before and after NCRT in predicting pCR in rectal cancer patients (37, 38). However, many tools and parameters are currently not up to a uniform standard.

In recent years, some inflammatory biomarkers and nutritional biomarkers can directly or indirectly respond to the inflammatory response and nutritional status of the body and have been found to be independent prognostic factors in patients with rectal cancer treated with NCRT (39–43). Most of the previous studies have focused on one or a few biomarkers, exploring their relationship with the efficacy of neoadjuvant chemotherapy in rectal cancer (44–48). However, it is clear that no single biomarker is currently powerful enough to achieve accurate prediction independently, and new models combining blood biomarkers and imaging parameters can achieve better outcomes. In this study, valuable blood biomarkers were filtered and combined with mrTRG to take advantage of the unique advantages of the different parameters as much as possible. A common feature of previous studies is the conversion of the obtained biomarkers from continuous variables to dichotomous variables, thus grouping patients in a simple way. However, it generates the problem of using different cut-off values in different studies. We did not perform simple dichotomization of the raw data in this study. It not only avoids the problem of various cut-off values due to sample differences, but also retains the advantage of continuous variables.

Our study has a few limitations. First, since this is a retrospective study with limited sample size, the possibility of selection bias during data collection cannot be excluded. Second, the blood biomarkers analyzed in this study are non-specific and may be influenced by various physiological or pathological factors. Hence, their values can vary over time. Nevertheless, our study focused only on the predictive role of these blood biomarkers prior to NCRT. Moreover, to analyze the efficacy of NCRT in treating rectal cancer, the final results should be tracked to determine long-term patient outcomes. In this study, the biomarkers’ long-term prognostic ability was not investigated. Thus, further large sample-sized studies are needed to determine these effects.

## Conclusion

This study extensively screened a variety of valuable pre-neoadjuvant blood biomarkers, such as CEA, NLR, LMR, and PFR, that could serve as predictors of pathologic complete regression and help improve the performance of MRI in predicting the efficacy of neoadjuvant therapy in patients with locally advanced rectal cancer. Combining pretreatment blood biomarkers with MRI metrics to create a clinical prediction model can effectively predict the efficacy of neoadjuvant therapy and thus help determine the optimal individual treatment regimen for LARC patients.

## Data availability statement

The original contributions presented in the study are included in the article/supplementary material. Further inquiries can be directed to the corresponding author.

## Ethics statement

The present retrospective study was approved by the Ethics Committee of the First Affiliated Hospital of Soochow University (Suzhou, China; approval no. 2022099), with a waiver of informed consent. Written informed consent for participation was not required for this study in accordance with the national legislation and the institutional requirements.

## Author contributions

SH: design and guidance, study supervision, and critical revision of the manuscript. XS: statistical analysis of the data, interpretation of the significance of the outcome, and manuscript drafting. MZ: software application, and production of partial charts. BS, GC, HY, JC, DW and WG: data acquisition. All authors have made contributions to this article and agree with the final version of the article.

## Funding

This work was supported by National Science Foundation (NSF) of Jiangsu Province of China grants (BK20191172), Project of Gusu Medical Key Talent of Suzhou City of China (GSWS2020005), and Project of New Pharmaceutics and Medical Apparatuses of Suzhou City of China (SLJ2021007).

## Acknowledgments

The author's thank all staff working in the department of general surgery in the First Affiliated Hospital of Soochow University, Soochow, China.

## Conflict of interest

The authors declare that the research was conducted in the absence of any commercial or financial relationships that could be construed as a potential conflict of interest.

## Publisher’s note

All claims expressed in this article are solely those of the authors and do not necessarily represent those of their affiliated organizations, or those of the publisher, the editors and the reviewers. Any product that may be evaluated in this article, or claim that may be made by its manufacturer, is not guaranteed or endorsed by the publisher.
